# Congruent audio-visual stimulation during adaptation modulates the subsequently experienced visual motion aftereffect

**DOI:** 10.1038/s41598-019-54894-5

**Published:** 2019-12-18

**Authors:** Minsun Park, Randolph Blake, Yeseul Kim, Chai-Youn Kim

**Affiliations:** 1 Department of Psychology, Korea University, Seoul, 02841 Korea; 2 Department of Psychology and Vanderbilt Vision Research Center, Vanderbilt University, Nashville, TN 37240 USA

**Keywords:** Human behaviour, Sensory processing

## Abstract

Sensory information registered in one modality can influence perception associated with sensory information registered in another modality. The current work focuses on one particularly salient form of such multisensory interaction: audio-visual motion perception. Previous studies have shown that watching visual motion and listening to auditory motion influence each other, but results from those studies are mixed with regard to the nature of the interactions promoting that influence and where within the sequence of information processing those interactions transpire. To address these issues, we investigated whether (i) concurrent audio-visual motion stimulation during an adaptation phase impacts the strength of the visual motion aftereffect (MAE) during a subsequent test phase, and (ii) whether the magnitude of that impact was dependent on the congruence between auditory and visual motion experienced during adaptation. Results show that congruent direction of audio-visual motion during adaptation induced a stronger initial impression and a slower decay of the MAE than did the incongruent direction, which is not attributable to differential patterns of eye movements during adaptation. The audio-visual congruency effects measured here imply that visual motion perception emerges from integration of audio-visual motion information at a sensory neural stage of processing.

## Introduction

Our everyday perceptual experiences arise from externally generated physical signals that concurrently activate our sensory systems, together creating rich, multi-dimensional impressions of salient objects and events. Moreover, the richness of these perceptual experiences can be accompanied by enhanced detectability of weak sensory events arising from two or more modalities (e.g., weak odors accompanied by visualization of the odor source) and by improved recognition of suprathreshold sensory events when those events are represented by information from two or more senses (e.g., speech comprehension when watching the speaker’s mouth). Over the past few decades, the number of studies characterizing this aspect of perceptual experience – multisensory interaction – has increased rapidly (for reviews, see^1,2^). In this paper, we focus on one particularly salient form of multisensory interaction: audio-visual motion perception^3,4^. Evidence for audio-visual interaction has been found with a variety of situations involving motion, including continuous motion^5–7^, biological motion^8–10^ and simple 2-frame/tone apparent motion sequences^11,12^.

There are numerous instances in the literature where watching visual motion influences what is heard when listening to auditory motion^12–16^. For example, when auditory apparent motion sequences – left vs right – are accompanied by simultaneously presented visual apparent motion sequences, perceivers are better at discriminating the auditory apparent motion direction when auditory sound is accompanied by the congruent visual apparent motion than when it is accompanied by the incongruent visual apparent motion^16^. By the same token, there are instances where auditory information influences the perception of visual motion^6,17–21^. Taken together, auditory and visual motion signals can influence each other when it comes to perceiving and judging motion information.

However, the patterns of results from previous studies are inconsistent in terms of the direction of influences between visual versus auditory information. While visual influences on auditory motion perception have been reported consistently as mentioned above, results are mixed in terms of auditory influences on visual motion perception^12,22,23^. Soto-Faraco *et al*.^12^, for example, showed that visual apparent motion equivalent in speed and direction to an auditory apparent motion event enhanced the ability to detect the perceived direction of auditory apparent motion. In contrast, auditory apparent motion did not bias judgments of perceived direction of visual apparent motion even when both forms of apparent motion were in the same direction. These asymmetric results might be explained by the modality appropriateness hypothesis wherein a greater weight is given to the sensory system with higher acuity for the task being performed^24,25^. It is generally believed that vision excels at registering precise spatial information while audition excels at registering precise temporal information (but also see^26^). Thus, the degree of influence of auditory versus visual information in a given task involving audio-visual stimulation may depend on the extent to which spatial information versus temporal information is critical, a point that has been made before^27^.

In a related vein, differences in task performance with unimodal versus bimodal testing do not perforce implicate audio-visual interactions at a *sensory stage* where afferent signals from the two modalities converge neurally. Evidence for bimodal interaction could also plausibly arise at a *stage* where auditory and visual sources of information are jointly evaluated to derive the likelihood of a given stimulus event. A textbook example of this idea is the ventriloquist effect, sound mislocalization that can be deftly explained by Bayesian-optimal combination of information arising independently from the eyes and ears^28^. In the case of motion perception, several studies have found that conjoint audio-visual motion stimulation yields improved motion detection performance whether or not the directions of auditory and visual motion are congruent^5,7,19^. The absence of direction-dependence points to facilitation in detection arising from probability summation of independent signals, i.e., improvement mediated by having two independent sources of information specifying motion. On other tasks involving motion, however, evidence shows that audio-visual judgments are affected by direction-dependent congruence between simultaneously presented auditory and visual motion (e.g.^29–31^), suggesting that audio-visual integration entails modification of the neural representations of direction at sensory level of processing.

To address this conundrum, in the present study we sought to develop a testing procedure wherein the perceptual task assessing the impact of concurrently presented auditory and visual motion did not itself involve audio-visual stimulation. To achieve this aim, we employed a purely visual task – judgment of direction of visual motion - whose performance was potentially dependent on the audio-visual context experienced immediately preceding task performance. Specifically, we deployed the visual motion aftereffect (MAE), i.e., the experience of illusory motion briefly seen following exposure (i.e., adaptation) to actual visual motion in a given direction^32^. The MAE can take several different forms, depending on the adaptation stimulus and the test stimulus. When adaptation entails translational motion in a given direction, e.g., rightward, a subsequently viewed stationary stimulus appears temporarily to move in the direction opposite to that of the adaptation motion, e.g., leftward^33^. Adaptation to translational motion can also produce an MAE when one views dynamic, Brownian-like random motion: such motion appears to have a net directional drift in the direction opposite to that of the adaptation motion^34^. Both forms of the MAE are commonly thought to reflect temporary reductions in neural activity within populations of visual neurons responsive to limited directions of motion^35^. Physiological evidence for such adaptation has been observed in neural responses measured from the early visual cortex of monkeys^36^ and humans^37,38^.

Is it plausible that sound could modulate neural adaptation of visually activated, direction-selective neurons at a sensory level? For several reasons, we think this possibility is plausible. First, we know that auditory motion can produce neural adaptation: exposure to an auditory stimulus that sounds like it is moving in a given direction can subsequently cause a stationary auditory stimulus to sound as if it were moving in the direction opposite to that of the previously heard sound, i.e., an auditory version of the MAE^39–41^. Second, it is well known that the magnitude of the visual MAE varies with the strength of the adapting motion, where strength refers to contrast, motion coherence, speed or duration. Is it plausible that sound, too, might modulate the strength of visual adaptation? This is not far-fetched, because we know that the MAE is influenced by other non-visual factors including imagination^42^ and attention^43–46^. Putting these facts together, we surmised that translational auditory motion and translational visual motion might indeed interact synergistically to impact the strength of a subsequently viewed visual MAE test stimulus. The following paragraph explains how this might happen.

If audio-visual sensory signals were to be combined when their directions and timing are congruent, the resulting MAE should be stronger if that bisensory combination transpires at or before the site of neural adaptation mediating the MAE. Integration of audio-visual signals, in other words, would produce stronger activity when those signals designate the same direction of motion, and that stronger activity would, in turn, produce a greater imbalance in activity among neurons responsive to direction of visual motion. Thus, we hypothesize that the visual MAE would be stronger and last longer when the direction of concurrent auditory motion was congruent with the direction of visual motion during adaptation, compared to conditions where auditory motion was absent or was incongruent with the direction of visual motion. This prediction would depend, of course, on selecting visual adaptation conditions (i.e., strength and duration) that themselves did not produce an asymptotic MAE.

**Figure 1:**
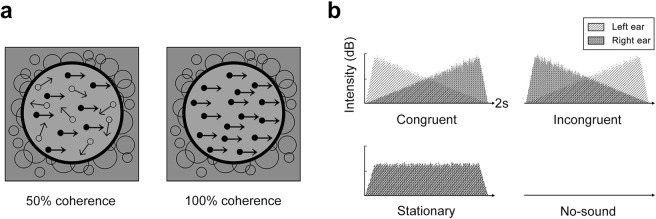
Schematic representation of the visual and auditory stimuli used during adaptation both in Experiment 1 and 2. (**a**) The visual stimulus was a RDK presented on a binocularly viewed video monitor. The fraction of dots moving coherently leftward or rightward (rightward in examples here) could be varied. In Experiment 1, RDKs with two different coherence levels were employed during adaptation, 50% and 100%. In Experiment 2, RDK coherence was fixed at 70% during adaptation. (**b**) The direction of auditory sounds was implemented by mimicking inter-aural intensity modulation, which was presented over binaural headphones. Considering sound and visual motion directions together, we created four different audio-visual conditions: congruent, incongruent, stationary, and no-sound.

To test this prediction, we performed two experiments in which the congruence of auditory and visual motion was varied during the adaptation phase of a trial and used the resulting MAE as an index of multisensory interaction. In an effort to tap into early-level sensory motion processing, we employed visual random-dot kinematograms (RDKs) moving either leftward or rightward (Fig. 1a), a class of visual animations widely used in the study of low-level motion perception^34,47,48^; the auditory stimulus was white noise in which the inter-aural intensity was continuously modulated to produce the perception of smooth, lateral sound motion (top panels of Fig. 1b). We specifically used repetitive, 2-sec pulses of concurrent visual and auditory motion to encourage the sense of the two events arising at the same time. Following a relatively brief period of adaptation to these concurrent audio-visual sequences, the resulting MAE was measured using either the duration of illusory motion of a stationary array of dots (Experiment 1) or the coherence of motion needed to nullify illusory drift in a subsequently viewed dynamic RDK (Experiment 2). So if auditory motion can indeed modulate the strength of visual motion, the visual MAE should take longer to dissipate, producing a longer MAE (tested in Experiment 1), and it would necessitate stronger actual visual motion to nullify (tested in Experiment 2). It is important to note that the MAE duration and intensity were being judged during test periods when auditory stimulation was not present – any influence of sound on the MAE must be attributed to its impact on the strength of the motion experienced during the prior period of *concurrent* audio-visual stimulation.

## Results

### Experiment 1. Audio-visual direction congruence’s effect on visual MAE duration

Twelve participants (ten naïve) completed a total of 32 trials during which they viewed a stationary test figure and reported the perceived direction and the duration of MAE (leftward or rightward) by pressing one of the two keys at the moment when the illusory motion ceased to be experienced. MAE strength was indexed by the duration of illusory motion^49^, which depends on the strength of the visual motion experienced during adaptation^32^. The test figure followed an adaptation period which entailed a total of 32-sec of actual exposure to visual motion (See Methods) during which the dynamic RDKs were presented with the directions (leftward, rightward) and coherence levels (50%, 100%) manipulated over blocks of trials. Over separate trials, we manipulated the audio-visual direction congruence based on the auditory stimuli (congruent, incongruent, stationary, no sound) accompanying the RDK. We predicted that the audio-visual direction congruence would strengthen the visual motion experienced during adaptation, resulting in longer MAE durations.

**Figure 2:**
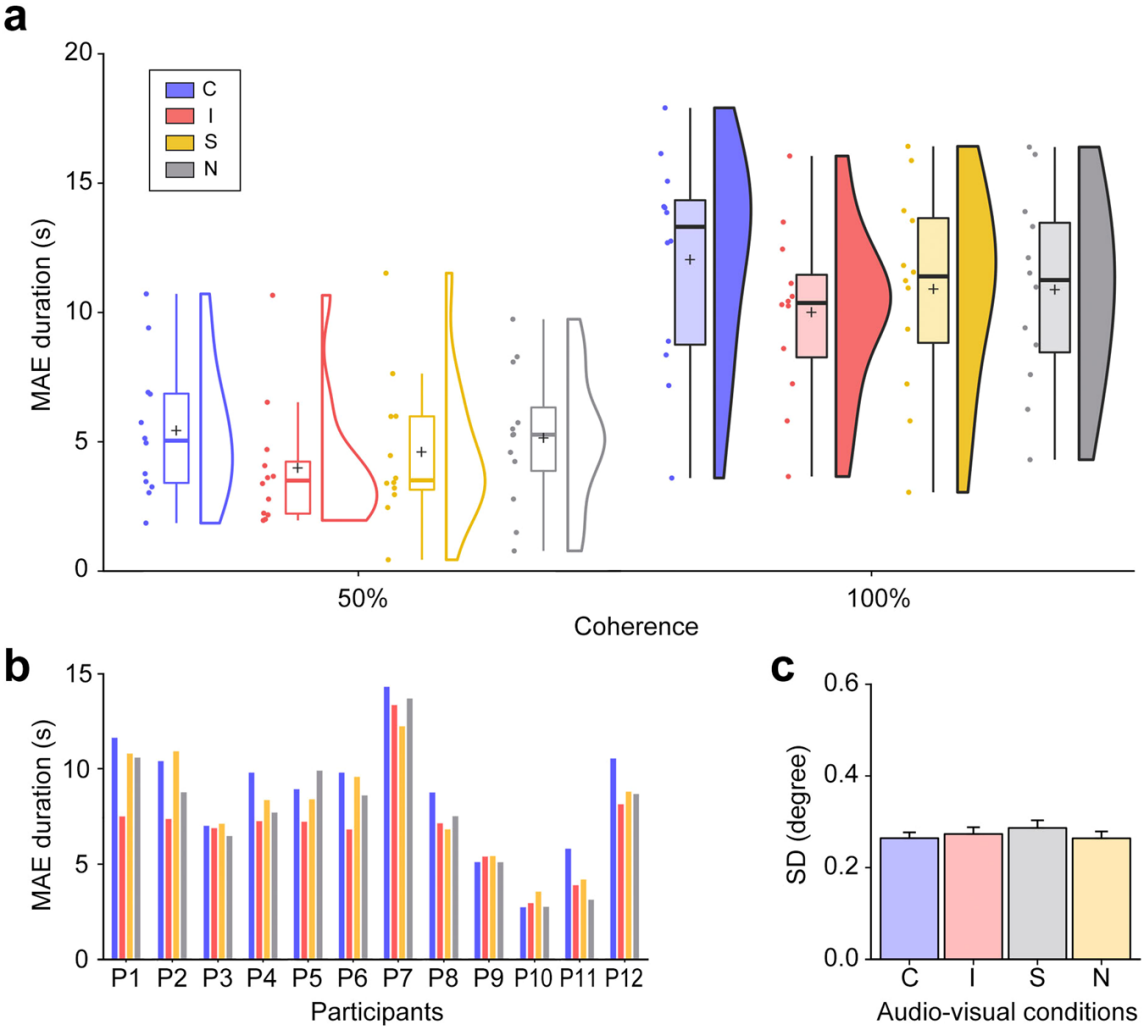
Results from Experiment 1. (**a**) Effect of audio-visual direction congruence during adaptation on the duration of resulting visual MAE. Rain cloud plots illustrate the estimated distributions of individual mean MAE duration in each coherence (50%, 100%) and audio-visual (congruent, C; incongruent, I; stationary, S; no-sound, N) conditions. Dots represent the individual mean MAE duration. The middle, horizontal lines on the box plots indicates the median and the lower and upper ends of the box indicates the first and third quartiles. The cross hairs indicate the mean. RDK directional motion accompanied by congruent sound during adaptation induced longer duration visual MAEs than did the stationary sound or the no-sound. RDK motion accompanied by incongruent directional sound during adaptation produced the briefest MAE of all conditions. This pattern of results was observed for both levels of adaptation motion strength, 50% and 100% coherence. (**b**) Individual data for audio-visual direction congruence. Individual participant data, including two of the authors (P1-MP, P4-RB), exhibited longer durations of visual MAE in the congruent condition than the incongruent condition. (**c**) Differences in standard deviation of the estimated distribution of left eye positions across audio-visual conditions. To evaluate the possibility that unstable eye fixation during adaptation affected the subsequent visual MAE, we compared the standard deviations of the estimated distributions of eye fixations for each audio-visual condition. Those comparisons revealed no statistically significant differences, implying that eye fixation during adaptation did not contribute to the results of Experiment 1.

As expected^44^, MAE durations differed between the two coherence levels (*F*(1, 11) = 58.40, *p* < 0.001, ${\eta }_{p}^{2}$ = 0.939, see Fig. 2a) and among the four audio-visual conditions (*F*(3, 33) = 8.47, *p* < 0.001, ${\eta }_{p}^{2}$ = 0.384, see Fig. 2a), after collapsing the data from the two visual motion directions that were not different statistically (*F*(1, 11) = 1.97, *p* = 0.188). Bayes factor (BF) analysis supported the audio-visual direction congruency effect on the perceived duration of visual MAE by the 95.297 of BF_10_, meaning that the current data were 95.297 times more likely to occur under the inclusion of the evidence for the factor of congruency than under the null hypothesis. The audio-visual direction congruency effect in the 50% coherence condition (i.e., congruent MAE duration > incongruent MAE duration) was not statistically different from that in the 100% coherence condition (*F*(3, 33) = 0.48, *p* = 0.699), thus allowing us to collapse the MAE duration data from the two coherence conditions for further examination of the source of the significant variances. Post hoc analyses of paired t-tests were performed with the false discovery rate (FDR^50^) correction applied for multiple comparisons of the six pairs of tests. The results showed that the MAE duration in the congruent condition was significantly longer than the MAE duration in the incongruent (*t*(11) = 4.34, *p* = 0.006 *FDR corrected*, Cohen’s d = 0.610; BF_10_ = 35.130, strongly favored) and in the stationary (*t*(11) = 3.35, *p* = 0.021 *FDR corrected*, Cohen’s d = 0.318; BF_10_ = 8.395, moderately favored) conditions. The difference between the congruent and the no-sound conditions also approached statistical significance (*t*(11) = 2.417, *p* = 0.051 *FDR corrected*, Cohen’s d = 0.245; BF_10_ = 2.224, anecdotally favored). In addition, the shorter duration of MAE in the incongruent condition approached statistical significance compared to the MAE duration in the stationary condition (*t*(11) = −2.106, *p* = 0.071 *FDR corrected*, Cohen’s d = 0.265; BF_10_ = 1.463, anecdotally favored) and in the no-sound condition (*t*(11) = −2.431, *p* = 0.051 *FDR corrected*, Cohen’s d = 0.390; BF_10_ = 2.268, anecdotally favored). There was no significant difference between the stationary and no-sound conditions (*t*(11) = −0.898, *p* = 0.388; BF_10_ = 0.404).

The pattern of audio-visual congruency effect was consistent across most individuals (Fig. 2b), and this pattern remained evident when MAE durations were reanalyzed with the data from the two non-naïve participants excluded (P1 and P4 in Fig. 2b). Results from the ten naïve participants showed that MAE durations were different across the coherence levels (*F*(1, 9) = 41.73, *p* < 0.001, ${\eta }_{p}^{2}$ = 0.931) and audio-visual conditions (*F*(3, 9) = 4.96, *p* = 0.007, ${\eta }_{p}^{2}$ = 0.264). The two-way interaction was not significant (*F*(3, 9) = 0.50, *p* = 0.686). In post hoc paired t-tests with the data merging two coherence levels, the MAE duration was significantly longer when audio-visual direction was congruent than when the direction was incongruent (*t*(9) = 3.623, *p* = 0.036 FDR corrected, Cohen’s d = 0.466) and stationary (*t*(9) = 2.599, *p* = 0.087 FDR corrected, Cohen’s d = 0.267).

The duration of motion adaptation was the same for all conditions, i.e., 32 sec taking into account the intermittent presentation of visual and auditory stimulation (see Methods). One might worry that this duration of adaptation promoted saturation of the MAE, thus invalidating its usefulness as an index of adaptation strength for the different conditions. Were this true, we would not expect to find differences between the no-sound condition and the other sound-related conditions. On the contrary, however, the congruent sound condition produced longer lasting MAEs than did the no-sound condition, and that was true for each of the participants. We are confident that ceiling effects are not unwittingly limiting the pattern of results in this Experiment.

The MAE is known to be confined to the region of the visual field, retinotopically defined, within which motion adaptation was experienced^51^, which suggests that inadvertent eye movements could differentially dilute the strength of the MAE across audio-visual conditions. The observed modulatory effect of audio-visual congruency during adaptation on the duration of visual MAE, however, does not stem from differential patterns of eye movements during the different audio-visual conditions. As shown in Fig. 2c, the standard deviations of the estimated (i.e., fitted with a normal distribution) left eye gaze deviation histograms across the four audio-visual conditions did not show a statistically significant difference (*F*(3, 30) = 1.11, *p* = 0.359).

Experiment 1 shows that audio-visual direction congruence during adaptation modulates the duration of the visual MAE. Audio-visual direction congruence during adaptation induces longer durations of the visual MAE. Conversely, the incongruence of audio-visual direction during adaptation abbreviates visual MAE durations. These modulatory effects of direction congruency did not differ between the two coherence levels of visual motion, indicating that audio-visual integration occurs effectively even when the direction of visual motion is unequivocally specified by visual information alone. This result seems contrary to the prediction based on inverse effectiveness whereby multisensory inputs are more likely to interact synergistically when the task-dependent sensory stimulus (vision, in this case) is relatively weak^52^, but it may well be attributable to the fact that even 50% coherence provides a sufficiently strong experience of coherent directional motion. To evaluate this possibility will require further testing of MAE strength using even weaker coherence levels. Overall, the findings from Experiment 1 imply that auditory motion information has a strong influence on visual motion processing during adaptation.

Could one argue that this direction-selective interaction arises from a residual auditory MAE generated when hearing apparently moving sound during visual adaptation and then experienced during visual MAE test phase? This seems very unlikely for the simple reason that there was no external sound source present during the test phase that could mediate an illusory impression of auditory sound. Sound’s only means of influence on the visual MAE would be through the prior, concurrent presentation of sound with visual motion during adaptation.

### Experiment 2. Audio-visual direction congruence’s effect on initial MAE strength

Systematic variations in the duration index of MAE strength in Experiment 1 implied the existence of significant audio-visual interactions. Those interactions might raise the possibility that participant’s awareness to the state of congruence between sound and RDK motions influenced the judgments of subsequent MAE, although it is unlikely that naïve participants figured out the purpose of the experiment during the experiment and translated it into their responses during test phases. Still, we felt it worthwhile to make expectation even less likely, so we repeated the experiment using a nulling procedure that measures a complementary index of MAE strength. Unlike MAE duration, the nulling procedure uses a brief test stimulus presented immediately following offset of the adaptation period, and it relies on a two-choice categorization judgment to derive the index of the strength of the resulting MAE.

**Figure 3:**
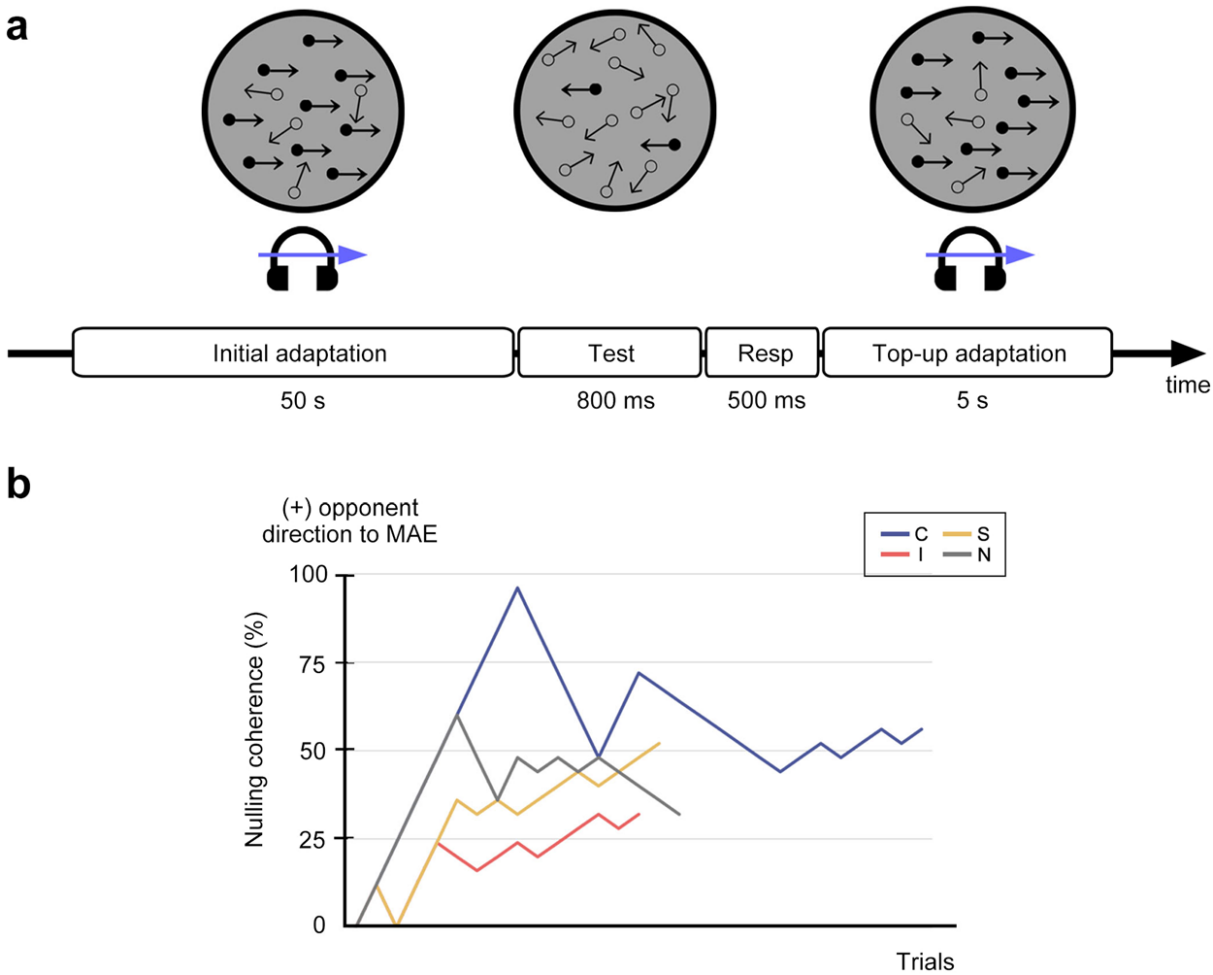
Procedures in Experiment 2 and an example of results from the adaptive staircase. (**a**) In a block, adaptation phase (50-s at the first trial and 5-s thereafter) and a brief test phase were alternated until the block was terminated once the randomly interleaved two staircases were completed. During the adaptation phase, the 70% coherence level of RDK was presented, and the directional sounds were either presented or not presented according to the audio-visual conditions (congruent, C; incongruent, I; stationary, S; no-sound, N). During the test phase, the direction and coherence level of the test RDK were determined by separate staircases designed to nullify the visual MAE. (**b**) An example of a staircase for one participant (P3-LHS in Fig. 4b). Staircases were terminated after nine reversals, and the nulling coherence derived from the staircase was calculated by averaging coherence values associated with the last six reversals.

For this experiment, we used dynamic RDKs presented during brief test phases inserted between periods of adaptation to 70%-coherence RDK motion (Fig. 3a). Using the procedure with two interleaved adaptive staircases (see representative results in Fig. 3b), the coherence of the RDK was varied to find the directional signal strength necessary to cancel any net motion impression engendered by motion adaptation^53^. This nullifying coherence level provides an index of MAE strength immediately following adaptation - stronger MAEs are evidenced by higher coherence levels needed to nullify the perceptually induced MAE. In this procedure, participants would be unable to know what the test stimulus actually portrayed and would have to rely on what they saw, not what they thought they were supposed to be seeing. Ten participants successfully completed this experiment.

**Figure 4:**
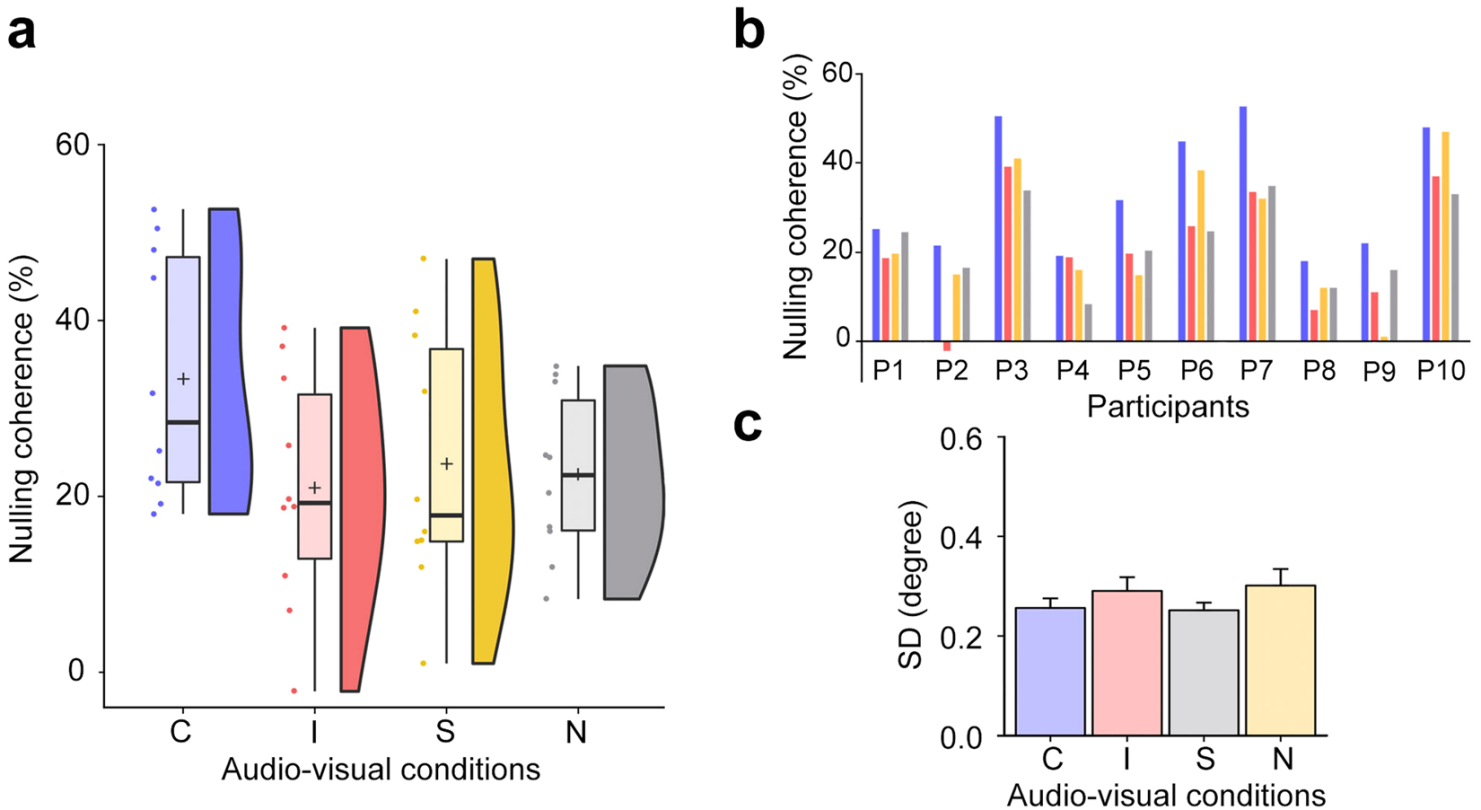
Results from Experiment 2. (**a**) Impact of audio-visual direction congruence on the strength of the dynamic visual MAE. The Rain cloud plots show the estimated distributions of the nulling coherence in each audio-visual condition. The absolute value of the nulling coherence indicates the intensity of the MAE and the polarity indicates the direction of signal dots needed to nullify the MAE. Thus, positive and non-zero numbers indicate the presence of the MAE. Individual mean nulling coherence values are presented as dots. Box plots illustrate the mean (cross hairs) and the median (bold, horizontal lines) as well as the first and third quartiles (each ends of the box) of the distribution. The nulling coherence values were significantly higher in the congruent than in the incongruent, stationary, and no-sound conditions. (**b**) The pattern of audio-visual direction congruence in individual data. Individual data, including the two authors (P1-MP, P2-RB), consistently showed higher coherence value to nullify the induced visual MAE in the congruent condition than the incongruent condition. (**c**) Differences of left eye positions during adaptation across audio-visual condition. Results comparing the standard deviations obtained from each histogram across audio-visual condition showed no statistically significant differences in eye gaze during adaptation. This implies that the effect of audio-visual direction congruence did not result from eye fixation instability during adaptation.

We expected that the congruence of the audio-visual direction would render visual motion stronger during adaptation, which requires higher motion coherence to nullify the induced MAE. As expected, the nulling coherence differed across the four audio-visual conditions (*F*(3, 27) = 10.70, *p* < 0.001, ${\eta }_{p}^{2}$ = 0.543, see the left panel of Fig. 4a). The BF analysis indicated that the strength of the evidence favoring the audio-visual direction congruency effect was 240.66 times more powerful than the evidence favoring the null hypothesis. Six FDR-corrected post hoc analyses of paired t-test showed that the nulling coherence in the congruent condition was significantly greater than the nulling coherence in the incongruent (*t*(9) = 5.90, *p* = 0.001 *FDR corrected*, Cohen’s d = 0.910; BF_10_ = 141.301, strongly favored), stationary (*t*(9) = 4.23, *p* = 0.004 *FDR corrected*, Cohen’s d = 0.666; BF_10_ = 21.311, strongly favored), and no-sound conditions (*t*(9) = 5.39, *p* = 0.001 *FDR corrected*, Cohen’s d = 0.914, BF_10_ = 81.312, strongly favored). However, there were no significant differences among incongruent, stationary, and no-sound conditions.

This strengthened MAE in the congruent condition was consistent in all the individual participants’ data (right panel of Fig. 4a). This pattern of audio-visual congruency effect was not affected even if the data were reanalyzed after excluding the two non-naïve participants (P1 and P2 in Fig. 4b), indicating that the group results did not emanate exclusively from the two participants. MAE nulling coherences were significantly different across audio-visual conditions (*F*(3, 21) = 11.01, *p* < 0.001, ${\eta }_{p}^{2}$ = 0.611). In post hoc analysis of paired t-test, the greater nulling coherence in the congruent condition was found than the nulling coherence in the incongruent (*t*(7) = 5.73, *p* = 0.002, Cohen’s d = 0.882), stationary (*t*(7) = 3.80, *p* = 0.014, Cohen’s d = 0.676), and no-sound (*t*(7) = 6.91, *p* = 0.001, Cohen’s d = 1.016) conditions. There were no significant differences in other comparisons among incongruent, stationary, and no-sound conditions. The differences in the MAE intensity across the audio-visual conditions are not attributable to differential eye movements during adaptation (Fig. 4c). The estimated standard deviations of the left eye gaze deviation histograms across four audio-visual conditions did not show a statistically significant difference (*F*(3, 24) = 0.54, *p* = 0.657).

Results from Experiment 2 indicate that the presentation of congruent audio-visual motion during adaptation strengthens the initial, perceived strength of the MAE. This strengthened MAE in the congruent condition was consistent in all the individual participants’ data (right panel of Fig. 4a). In Experiment 2, the motion coherence and the direction of test stimuli were controlled by the staircase procedure on each trial, making it very unlikely that participants’ expectations would be involved in their response. The advantage of the method used in Experiment 2 mitigated the factors that may affect the decision stage of audio-visual motion processing, thereby convincing us that the observed effect of audio-visual direction congruence arose at the perceptual stage of the processing. The boosted effect of the audio-visual direction congruence replicates the results obtained using MAE duration following adaptation during the congruent condition in Experiment 1. Again as a reminder, sound’s only means for influencing the visual MAE is through its impact on the strength of the visual motion experienced during concurrent audio-visual stimulation during the adaptation period.

What is different between results for the two experiments is the effect of incongruent sound on the strength of the MAE: in Experiment 1, incongruence in sound and RDK motion direction reduced MAE duration relative to no-sound and stationary sound conditions, but in Experiment 2 the RDK motion strength required to nullify the MAE was not different than the nulling coherence associated with the stationary and the no-sound conditions. Why might that be? The answer may well have to do with the fact that the two measures of MAE strength – duration and nulling coherence – tap into different aspects of visual adaptation. Specifically, the coherence required to nullify the MAE gauges the initial strength of adaptation immediately following offset of the adapting motion whereas the duration of the MAE gauges the decay of adaptation’s aftereffect over time. We have no way to insure that initial strength of adaptation was equivalent in the two experiments and, therefore, whether the start-points of the decay of adaptation was the same for both (see^54^ for a model that explicates the distinction between decay and nulling). This could provide some basis for the differential effect of incongruent sound during adaptation on the decay-based MAE and the nulling-based dynamic MAE. What we can say for certain is that the initial strength and decay time were amplified by presentation of congruent sound relative to presentation of incongruent sound, stationary sound or no sound.

## Discussion

We found that adaptation to translational visual motion results in an initially stronger and longer lasting MAE when that visual adapting motion is accompanied by sound moving in the same direction as that visual motion, compared to sound motion in the opposite direction or, for that matter, to stationary sound or no sound at all. Before considering the potential implications of our results, we want to acknowledge that ours is not the first study to exploit adaptation aftereffects to examine multisensory interactions. Deas, Roach, and MacGraw^55^ reported that adaptation to translational visual motion caused the perceived location of a subsequently heard, stationary sound to be displaced in a direction opposite that of the visual motion. While not a conventional MAE – the stationary sound did not seem to move – the displacement of the sound’s perceived location clearly does reveal an impact of visual motion adaptation on auditory perception. In a similar vein, Hidaka and colleagues^56^ found that the net direction in the perceived flow of a weakly coherent RDK was biased when accompanied by a stationary tone previously paired with strongly coherent visual motion, i.e., a visual MAE contingent on audition. Kitagawa and Ichihara^57^ used loudness-modulated sound paired with a size-modulated visual square to generate an auditory aftereffect (illusory change in auditory loudness) and a visual aftereffect (motion depth produced by illusory changes in perceived visual size). They found that congruent audio-visual adaptation (e.g., increasing loudness paired with increasing size) induced a stronger auditory aftereffect than did auditory adaptation alone, but the magnitude of the visual aftereffect was not modulated by the sound. Finally, Berger and Ehrsson^58^ found that adaptation to a horizontally moving sound could generate illusory translational flow in a subsequently viewed visual RDK, i.e., a cross-modal adaptation aftereffect. Considered together, results from those studies, together with the present findings, underscore the fruitfulness of adaptation aftereffects as a tool for examining audio-visual interactions.

At the same time, several previous studies have failed to find a robust impact of adaptation to auditory motion on subsequent visual motion perception. As mentioned above, Kitagawa and Ichihara^57^ found that adaptation to sound that was actually increasing or was actually decreasing was unable to produce a subsequent visual aftereffect of motion in depth. Similarly, Jain and colleagues^23^ adapted participants to moving sound to see if that would produce a cross-modal visual MAE, which it did not. In both instances, in other words, sound adaptation did not impact subsequently viewed visual motion. We, however, have found that exposure to sound during adaptation potentiates visual adaptation, as evidenced by stronger and longer MAEs when visual and auditory motion directions were congruent (see also^58^). What might account for these seemingly inconsistent findings? One possibility is that the auditory stimuli in some of those previous studies relied on sound whose direction was implicitly connoted (e.g., rising/falling loudness denoting approaching or receding sound), whereas the sound motions in our study were defined explicitly by interaural intensity differences. But this cannot be the entire reason, for Jain *et al*.^23^ also used interaural loudness to create sound motion yet failed to find that this sound motion was able to induce a visual MAE on its own. That result, of course, is not inconsistent with our findings because we did not test for an auditory MAE in our study. It remains to be learned why there exists an asymmetry in the adaptation strengths of visual motion and auditory motion.

Turning now to the implications of our results, our findings provide evidence for genuine integration of visual and auditory motion signals at a stage of processing sufficient to impact visual motion adaptation. The logic of our experimental design focuses on neural synergy during the adaptation period, not during the testing period when the weak or ambiguous motion was presented. To presume that judgments of visual motion direction during the MAE test period could be directly impacted by sound presented before the visual test stimulus even appeared would violate one of the two pillars of multisensory interaction, namely the temporal contiguity principle stipulating that sound stimuli and visual stimuli occur contiguously if they are to interact synergistically^59^. Thus we conclude that the critical interactions prompting the audio-visual integration we have documented occur during the adaptation period, when sound and visual motion were presented in close spatial and precise temporal coincidence. We interpret our results as evidence for genuine neural confluence of sensory signals arising from auditory and visual modalities at a neural locus where patterns of activity among direction-selective visual neurons arise, patterns of activity that register the direction of translational visual motion^38^. In the context of our experimental paradigm, integration of independent streams of auditory and visual information seems inadequate to account for the MAE results. To be sure, this does not mean that cue combination (e.g., Bayesian inference) does not transpire in other conditions, such as situations where neural information associated with weak signals in one modality are biased by stronger, more reliable signals in a second modality^5^.

Taken together, our main finding implicates integration of neural signals arising within auditory and visual pathways, resulting in enhanced neural responses within bimodal neurons. Neurophysiological evidence points to multiple sites where such interactions might occur, including deep layers of the superior colliculus (SC)^52,60,61^ and cortical areas interconnected to the SC including the middle temporal (MT) area^62^ where, it is well known, adaptation produces temporary changes in neural activity within direction-selective cells^32,63^. Studies in humans using fMRI also show that activation in human V5/MT + area is modulated by the auditory motion. Thus, for example, lateral sound motion induces increased blood oxygenated level dependent (BOLD) signals in area of V5/MT + when moving sound was accompanied by visual motion^64^ or when moving sound was heard while participants were blindfolded^65^. Of particular relevance to our audio-visual congruency effect, Scheef and colleagues^66^ demonstrated enhanced activation in V5/MT + in the presence of congruent audio-visual motion stimuli compared to incongruent audio-visual or unimodal motion stimuli. These neurophysiological, electrophysiological and fMRI data are congruent with our psychophysical findings pointing to involvement of audio-visual neural integration when viewing motion while simultaneously hearing sound congruent with that motion.

## Methods

### Participants

Thirteen and fourteen participants took part in Experiment 1 and 2, respectively. Both Experiments included two of the authors (MP and RB). Experiment 1 was conducted at Vanderbilt University (VU) and Korea University (KU), and Experiment 2 was performed at KU except for one participant (RB at VU). All participants had normal or corrected-to-normal visual acuity. Except for the two authors, all the participants were naïve to the purpose of the experiment. They gave informed consent approved by the Institutional Review Board of VU (IRB #000700) and KU (IRB 1040548-KU-IRB-17-85-A-1) prior to the experiment. All procedures were approved by the Vanderbilt University Human Research Protection Program or Institutional Review Board of Korea University, and the study was carried out in accordance with the Code of Ethics of the World Medical Association (Declaration of Helsinki).

### Apparatus and stimuli

Gray-scale visual stimuli were displayed on a gamma-corrected CRT monitor (frame rate 100 Hz), which was used both in VU and KU (viewing distance 108 cm in VU, 95 cm in KU). Binaural auditory stimuli were delivered via headphones. Participants viewed the monitor with their head stabilized by a head/chin rest. The position of their left eye was tracked at a sample rate of 500 Hz using an Eyelink eye tracker (Model 1000, SR Research), controlled by the Eyelink toolbox in MATLAB^67^. The experiment was conducted in a quiet dark room.

Auditory stimuli were created, manipulated and saved using Sound Studio (v. 4.9.3, Felt Tip Inc.). Those sounds were stored as.wav files on the computer and utilized by MATLAB for presentation over binaural headphones (Yamaha YH-1 over-ear; 150 ohm impedance, 96 dB/mW). Each discrete sound comprised a 2-s pulse of white noise generated at a sampling rate of 44.1 kHz. Auditory motion was simulated by using a linear ramp to cross-fade the intensity (dB) of the white noise samples presented to left- and right-channels of the headphones. This produced a clear impression of sound movement either leftward or rightward depending on the leading ear of the cross-fade. For the stationary sound, white noise was played at constant, equal intensity over both channels of the headphones. The initial onset and final offset of all sounds were ramped on and off (linearly in units of dB) over 150 ms, to preclude abrupt transients.

Visual stimuli used in the adaptation phase were RDKs created by MATLAB using Psychophysics Toolbox-3^68,69^. RDKs appeared within a light gray (VU: 62 cd m^2^, KU: 28 cd m^2^) circular aperture 4° in diameter, containing 240 black dots (dot size: 4.74 arcmin; dot density: 19 dots/deg^2^) and a central red fixation point. The RDK dots were centered within a 6° diameter, circular background pattern filled with outline “bubbles” of different sizes. The dots began their motion trajectories from quasi-random positions within the aperture, and they were repositioned by 1 pixel every 10 ms. The dots moved at a fixed speed of 2°/s, which matched the duration required for a dot to move across the entire diameter of the RDK window during the 2-s auditory noise presentation. Signal dots moved coherently either leftward or rightward; noise dots selected their directions randomly. The mean lifetime of a given dot’s trajectory was 400 ± 200 ms. At the end of its lifetime or upon moving outside of the aperture, a given dot was ‘reborn’ at a new random position.

As with the auditory stimuli, the dots appeared and disappeared smoothly with their luminance modulated gradually for 150 ms at the beginning and at the end of the presentation. This procedure was implemented to prevent abrupt stimulus onsets and offsets that could potentially generate motion energy^70^. During the test phase in Experiment 1, stationary dot patterns were used to measure the duration of MAE. In Experiment 2 where we measured a nulling coherence level, dynamic RDKs were used to vary perceived motion coherence over trials. Mean lifetime of test RDKs was relatively brief (16 ± 40 ms).

### Procedures

#### Experiment 1

Each trial started with a 40-s visual motion adaptation phase accompanied by sounds of one of the four audio-visual conditions. During the adaptation phases with sound, the 2-s sound was played 16 times with an 800-ms interval interleaved, and the onset of RDK was synchronized with the sound. To encourage attention to both the auditory and the visual motion, participants were required to perform a simple attention task associated with each relatively brief pulse of visual motion: the duration of RDK was either 1.8 s or 2.2 s, and participant reported whether the duration of RDK was shorter or longer than that of the accompanying sound. On trials without sound, simply the duration of the RDK was reported (short or long). Prior to the main session, participants underwent a practice session to familiarize them with the attention task; this practice was continued until their performance achieved or exceeded 70% accuracy. Based on the attention task performance, we excluded one participant, thus a total of twelve participants were included for the final analysis.

After the adaptation phase, a stationary pattern appeared within the circular stimulus window. Participants were instructed to press one of the two keys corresponding to the perceived MAE direction the moment the dots ceased their illusory motion. For the trials where participants did not experience any MAE, a third key-press was made. Upon this key-press, an enforced, 15-s break was inserted with a stationary dot pattern and a stationary white noise sound before the next trial was launched. If the participant experienced even a hint of visual MAE after the break, they pressed the spacebar to introduce an additional 10-sec recovery period, and this procedure continued until the MAE had dissipated completely.

Participants were instructed to maintain strict fixation on the central fixation point. Horizontal and vertical positions of the participant’s left eye were monitored during the adaptation phase using the eye tracker. Eye calibration and validation were repeated every 8 trials.

In the main session, participants performed a total of 32 trials: 2 visual directions (leftward, rightward) × 2 coherence levels (50%, 100%) × 4 audio-visual conditions (congruent, incongruent, stationary, no-sound) × 2 repetitions (Fig. 1). The order of the trials was pseudo-randomized. The main session took about an hour.

#### Experiment 2

In the practice session, participants were familiarized with the appearance of global net flow from RDKs. On each self-paced practice trial, either a leftward or rightward RDK was presented for 800 ms. Initial coherence level was 70% and thereafter it was controlled by 1-up 2-down staircase method to find the threshold value supporting 71% correct performance^71^. Participants reported the global direction of the RDK by pressing one of the two keyboard buttons, followed by auditory feedback. Four successive staircases were performed. The fourteen participants exhibited coherence thresholds ranging from 6% to 50.2% (22.9% ± 13.8%), values well below the level of coherence used for adaptation during the main experiment.

The main session comprised of a series of the adaptation and the test phases administered in a block design (Fig. 3a). Each block began with an initial 50-s adaptation period followed by a test phase. A 5-s top-up adaptation then followed each test phase thereafter. During the adaptation phase, a 70%-coherence RDK was displayed for 2 s simultaneously accompanied by 2-s sound, and they were repeatedly presented with a 500-ms interval. Participants were instructed to attend to both auditory and visual stimuli with fixation maintained on a central red fixation dot. Following the adaptation phase, the test RDK was presented for 800 ms. The coherence and the direction of the test RDKs were controlled by two independent 1-up/1-down staircases randomly interleaved. The coherence level at the first test trial was 0%. Step size of the coherence level was 12% for the initial three reversals and 4% thereafter. Following each RDK test presentation, participants reported the perceived global direction of the test RDK by pressing one of the two keyboard buttons within 500 ms after the test RDK disappeared. When they failed to respond during the test phase (which rarely happened), the coherence level in that trial was presented again on the next test presentation. Each of the two staircases terminated after nine reversals, and the mean of the last six reversals was taken as the nulling coherence of the corresponding staircase. A test block terminated after both interleaved staircases for that condition had reached the ninth reversal points. Participants took a 4-min break between blocks. If there was any residual MAE perceived on the stationary dot patterns after the break, participants took an additional 30-s break until all hints of coherent motion had disappeared. The stability of the left eye was monitored by eye tracking throughout the main session. Eye calibration was performed before every block.

Each audio-visual condition included four staircases (Fig. 3b). The nulling coherence value for each condition was estimated by averaging the coherence values obtained from the four staircases. The absolute value of nulling coherence value indicates the intensity of the MAE and the polarity indicates the direction of signal dots needed to nullify the MAE. Positive numbers denote the direction that was opposite to the direction to the MAE for nullifying, which indicates the presence of the MAE. Four participants who did not experience MAE in the no-sound condition during session 1 (i.e., nulling motion of zero coherence or negative polarity of nulling direction) were not tested on the remaining conditions, leaving a total of ten participants who completed this experiment.

Each of two sessions comprised four blocks, each of which was randomly assigned to one of the four audio-visual conditions. Each participant was visually adapted to only one of two directions (left or right) throughout the experiment, with the adaptation direction counterbalanced across participants. The experiment, including practice, main sessions and breaks, took approximately 2.5 hours.

### Analysis

#### Bayes factor analysis

To quantify the evidence for the hypothesis that audio-visual congruency modulates the strength of visual MAE, we conducted Bayes factor (BF) analysis using the JASP software^72^. We calculated the ratio of Bayesian probability that the current data favor the null hypothesis (no difference of the MAE strength according to the audio-visual congruency) to the probability that they favor the alternative hypothesis. For all BF analysis, we used a common Cauchy prior with width 0.707. The BF_10_ value signifies how strongly support the alternative hypothesis than the null hypothesis.

#### Gaze stability analysis

We analyzed the horizontal eye positions of participants’ left eye during adaptation phase. Recorded horizontal left eye position samples were transformed to deviations from fixation, and the number of deviations was reduced by averaging the deviations within every 20-ms bin. By creating a gaze histogram for each condition using these deviation samples, we extracted a parameter of standard deviation from the histogram fitted with a normal distribution.

## Data Availability

The datasets of Experiments 1 and 2 are available on https://osf.io/tfec5/.
